# Integrating spatial transcriptomics and single-cell RNA-sequencing reveals epithelial cell alterations in benign prostatic hyperplasia following finasteride treatment

**DOI:** 10.1016/j.gendis.2025.101526

**Published:** 2025-01-10

**Authors:** Yanting Shen, Xiawei Fei, Liyang Dai, Ting Zhu, Jican Liu, Zhenqi Wu, Huan Xu, Huifeng Li, Zhong Wang

**Affiliations:** a Department of Urology and Andrology, Gongli Hospital of Shanghai Pudong New Area, Shanghai 200135, China; b Department of Urology, Qingpu Branch of Zhongshan Hospital Affiliated to Fudan University, Shanghai 201700, China; c Department of Pathology, Qingpu Branch of Zhongshan Hospital Affiliated to Fudan University, Shanghai 201700, China; d Department of Urology, Shanghai Ninth People's Hospital, Shanghai Jiaotong University School of Medicine, Shanghai 200011, China

The pathogenesis of benign prostatic hyperplasia (BPH) is commonly regarded as androgen-dependent. The first FDA-approved androgen-targeted medication for BPH, finasteride, achieves its therapeutic effect by selectively inhibiting type II 5-alpha reductase (SRD5A2). This inhibition reduces the androgenic response by attenuating the interaction between dihydrotestosterone and androgen receptor (AR), ultimately leading to a reduction in prostate volume and alleviation of BPH-associated symptoms. However, it is noteworthy that non-response to finasteride may develop in certain BPH patients,^1^ indicating a potential inadvertent promotion of disease progression by this treatment. Nevertheless, the underlying mechanism remains elusive. Recent researches suggest that it may be associated with alterations within the prostate gland epithelia.^2^ Therefore, we designed this study to elucidate this phenomenon by employing spatial transcriptomic (ST) and single-cell RNA sequencing (scRNA-seq) (methods were described in Supplementary Materials in detail). Our findings will improve treatment adjustments by enabling a more accurate evaluation of patient's response to finasteride therapy, thus enhancing therapeutic outcomes.

Three BPH tissues obtained from one patient treated with finasteride (Fin-P) and two untreated patients (Fin-N) were subjected to ST sequencing (Table S1). A total of 6136 ST spots were acquired and classified into eleven clusters based on unique expression patterns of specific genes (Fig. S1; Fig. 1A). The cell type of each cluster was identified using robust cell type decomposition methods (Fig. S2). As shown in Figure 1B, C2 and C3 clusters predominantly comprised epithelia, as indicated by their relatively higher epithelial dentate weight and lower stromal and immune dentate weights. Additionally, the well-recognized epithelial marker gene, *EPCAM* (epithelial cell adhesion molecule), was used to precisely identify epithelial ST spots within C2 and C3 clusters for subsequent analysis.

**Figure 1:**
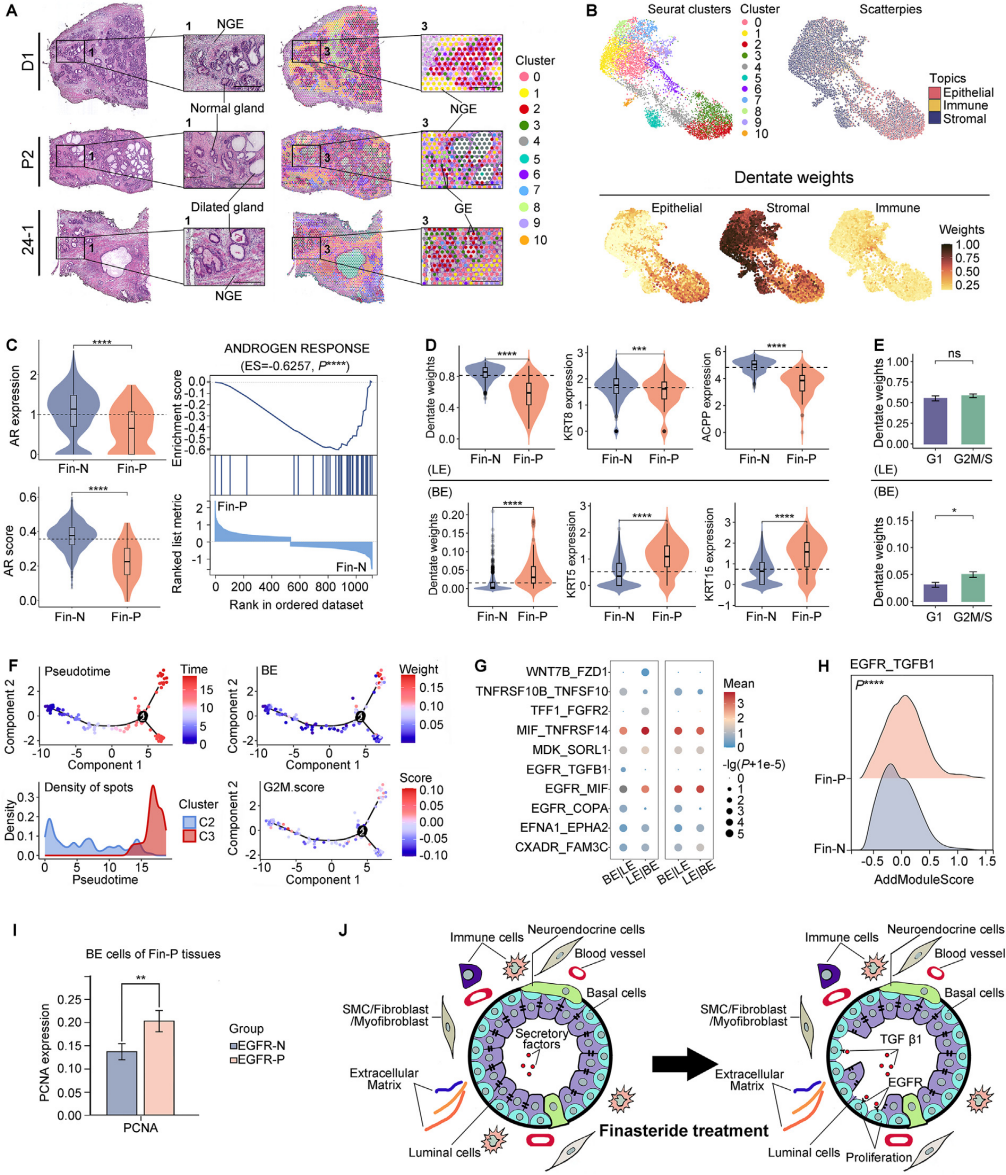
Integrating ST and scRNA-seq reveals epithelial cell alterations in BPH following finasteride treatment. **(A)** The images depicting hematoxylin-eosin-stained sections and BPH tissue sections colored by clusters. **(B)** The UMAP plot showing cell type decomposition in each histological structure (robust cell type decomposition methods): Seurat clusters (upper left); dentate weights of epithelial, stromal, and immune cells (bottom); the scatterpies illuminating cell-type deconvolution for each spot (upper right). **(C)** The violin plots depicting the *AR* expression level and AR-mediated transcriptional gene set score (AR score) (left) and the Gene Set Enrichment Analysis (GSEA) plot interpreting changes in the expression levels of hallmark androgen response gene set (right). **(D)** The violin plots depicting the LE and BE cell dentate weights and the expression of *KRT8*, *ACPP*, *KRT5*, and *KRT15*. **(E)** The bar plots depicting the LE and BE dentate weights in Fin-P epithelia in the G2M/S phase compared with those in the G1 phase. **(F)** Pseudo-time cell trajectory of the Fin-P epithelial ST spots colored by the pseudo-time, BE cell dentate weights, densities of spots in each cluster, and G2M score. **(G)** The dot plots depicting the significantly expressed ligand-receptor (L–R) pairs between LE and BE cells in Fin-P tissues (CellphoneDB). **(H)** The ridge plot depicting the co-expression score of *TGFB1* and *EGFR* in Fin-P and Fin-N epithelial ST spots. **(I)** The bar plot depicting the expression of *PCNA* in Fin-P *EGFR*-positive (EGFR-P) and *EGFR*-negative (EGFR-N) BE cells. **(J)** Alterations and the underlying mechanism in prostate epithelial cells following finasteride therapy. (A–F, H) shows ST data and (G, I) shows scRNA-seq data. *P*-values for the comparison between two variables were determined using a two-sided Wilcoxon rank-sum test. ∗*P* < 0.05; ∗∗*P* < 0.01; ∗∗∗*P* < 0.001; ∗∗∗∗*P* < 0.0001. ST, spatial transcriptomic; scRNA-seq, single-cell RNA sequencing; BPH, benign prostatic hyperplasia; BE, basal epithelial; LE, luminal epithelial.

Several studies indicate that finasteride treatment may result in a reduction or loss of SRD5A2 in BPH tissues, potentially leading to off-target effects and counteracting its inhibitory impact on androgen response.^1^ However, after analyzing the BPH epithelial ST spots, we found no evidence supporting a decrease in *SRD5A2* expression in Fin-P versus Fin-N epithelia (Fig. S3). Additionally, the expression levels of *AR* and classical AR-mediated transcriptional genes such as kallikrein-related peptidase 2 (*KLK2*) and kallikrein-related peptidase 3 (*KLK3*), as well as scores for AR-mediated transcriptional gene set and hallmark androgen response gene set, were found to be significantly lower in Fin-P versus Fin-N epithelia (Fig. 1C; Fig. S4, 5). scRNA-seq analysis consistently yielded concordant results (Fig. S6). These findings suggest that finasteride treatment effectively attenuates AR-dependent transcriptional activity in BPH epithelia, without affecting the expression of SRD5A2.

The normal prostate tissue typically has an equal ratio of basal epithelial (BE) and luminal epithelial (LE) cells. However, our BPH ST findings revealed a significant reduction in LE cells and an increase in BE cells within Fin-P epithelia versus Fin-N epithelia (Fig. 1D). We assume that the reduction of LE in Fin-P epithelia may result from the attenuation of AR-mediated transcriptional activity, which suppresses androgen response, leading to decreased support for cell growth^2^ and increased apoptosis.^3^ To substantiate it, we initially conducted a comparative analysis of the hallmark apoptosis gene set scores between Fin-P and Fin-N BPH epithelia. The results clearly showed that Fin-P epithelia had significantly higher scores than Fin-N epithelia (Fig. S7). Subsequently, employing correlation analysis, we observed that attenuated androgen response significantly enhanced apoptosis and intensified LE cell loss in Fin-P BPH epithelia (Fig. S8A). Additionally, analysis of the LE to BE cell ratio using BPH scRNA-seq datasets yielded consistent results with ST analysis (Fig. S8B). Pseudo-time cell trajectory analysis on Fin-P LE cells revealed a developmental trajectory showing the transition from high to low AR-mediated transcriptional activity. Along a specific branch of this trajectory (cell fate 2), expression levels of proapoptotic genes *BAX* (BCL2 associated X) and *BID* (BH3-interacting domain death agonist) were significantly up-regulated in LE cells (Fig. S8C). This up-regulation was accompanied by down-regulation of *KLK2* and *KLK3*. Collectively, these findings indicate that finasteride therapy promotes apoptosis by attenuating AR-mediated transcriptional activity, which may be the primary factor contributing to LE loss.

The higher proportion of BE cells in Fin-P BPH epithelia may mainly stem from their increased proliferation. Analysis of the Fin-P epithelial ST spots revealed a significant increase in the BE dentate weights during the actively proliferating G2M/S phase compared with the relatively quiescent G1 phase, while no noticeable findings for LE were observed (Fig. 1E). Furthermore, the pseudo-time cell trajectory analysis demonstrated a positive correlation between BE dentate weights and the G2M score, indicating enhanced cell proliferation with an increase in BE cells (Fig. 1F). Consistently, Spearman correlation analysis revealed significant positive correlations between G2M score, S score, and GOBP cell cycle gene set score with BE dentate weights (Fig. S9A). However, no similar positive correlations were observed between LE dentate weights and these variables (Fig. S10). Additionally, BPH scRNA-seq datasets analysis corroborated these findings by revealing a significant up-regulation in G2M scores and gene expression levels of proliferating marker gene *PCNA* (proliferating cell nuclear antigen) in Fin-P BE cells versus Fin-N BE cells (Fig. S9B, C). These findings suggest that finasteride treatment can enhance BE proliferative activity.

The mechanisms driving BE proliferation in Fin-P BPH tissues remain poorly understood. Previous studies suggest that attenuated AR-transcriptional activity may impact epithelial barrier function, creating favorable conditions for factors secreted by LE cells to affect BE cells.^4^ Our analysis of BPH scRNA-seq and ST data showed an up-regulation of the *TGFB1_EGFR* pair in Fin-P epithelia, which positively correlated with G2M and GOBP cell cycle gene set score (Fig. 1G, H; Fig. S11A, B). Additionally, BPH scRNA-seq datasets analysis demonstrated that epidermal growth factor receptor (*EGFR*) expression was significantly higher in Fin-P BE cells than in Fin-N BE cells (Fig. S11C), and *EGFR*-positive BE cells exhibited a significantly higher *PCNA* expression level than *EGFR*-negative BE cells in Fin-P tissues (Fig. 1I). Considering the well-documented pivotal role of EGFR in driving cell proliferation across diverse cancer types,^5^ we infer that the activation of EGFR on BE cells, stimulated by specific factors such as transforming growth factor-beta 1 (TGF-β1) secreted by LE cells, may potentiate BE cell proliferation.

In conclusion, our findings indicate that following finasteride treatment, AR-mediated transcriptional activity is significantly attenuated in BPH epithelia (Fig. 1J). This attenuation leads to persistent inhibition of androgen response, resulting in LE cell apoptosis and loss. Consequently, the integrity of the epithelial barrier is compromised, leading to enhanced secretion of pro-inflammatory factors like TGF-β1 into peri-glandular spaces. These factors may further facilitate BE cell proliferation through activating EGFR. Inconsistent with previous studies,^4^ our findings highlight the crucial role of EGFR in BE cell proliferation, emphasizing the potential involvement of BE cells in the non-response or inadequate response observed following finasteride treatment. Our findings suggest that targeting EGFR on BE cells offers a promising AR-independent therapeutic approach for BPH patients who exhibit no response or incomplete response to finasteride.

## Ethics declaration

The study was approved by the Ethical Committee of QingPu Branch, Zhongshan Hospital, Shanghai, China (approval number: Qingyi2020-24).

## Funding

This study is supported by the National Natural Science Foundation of China (No. 62101319), the Qingpu District Health Commission Discipline Talent Project of Shanghai, China (No. YY2023-3, TX2023-4), and the Medical Discipline Construction Project of Pudong New Area Health System (Shanghai, China) (No. PYWgf2021-06).

## CRediT authorship contribution statement

**Yanting Shen:** Writing – review & editing, Writing – original draft, Methodology, Investigation, Data curation, Conceptualization. **Xiawei Fei:** Writing – review & editing, Writing – original draft, Methodology, Data curation, Conceptualization. **Liyang Dai:** Methodology, Data curation. **Ting Zhu:** Methodology, Data curation. **Jican Liu:** Methodology, Investigation, Data curation. **Zhenqi Wu:** Investigation, Data curation. **Huan Xu:** Writing – original draft, Methodology, Investigation, Data curation, Conceptualization. **Huifeng Li:** Writing – original draft, Methodology, Data curation, Conceptualization. **Zhong Wang:** Writing – review & editing, Methodology, Investigation, Data curation, Conceptualization.

## Data availability

The raw ST data were deposited in the NCBI Gene Expression Omnibus (GEO) database.

## Conflict of interests

The authors declared no competing financial interests.
