# Supplementary material for: Integrating spatial transcriptomics and single-cell RNA-sequencing reveals epithelial cell alterations in benign prostatic hyperplasia following finasteride treatment
Source: Genes Dis. 2025 Jan 10;12(5):101526. doi: 10.1016/j.gendis.2025.101526 (PMC12166679; doi:10.1016/j.gendis.2025.101526)
Supplement: Multimedia component 1 [file mmc1.docx]

**Supplementary Materials**

**Materials and methods**

**Study design**

We conducted this study using spatial transcriptomic (ST) and single-cell RNA sequencing (scRNA-seq). Initially, three prostate tissue samples were obtained from different patients diagnosed with benign prostatic hyperplasia (BPH) at the QingPu Branch of Zhongshan Hospital in 2019. These samples underwent ST sequencing. Among them, one sample was collected from a BPH patient who had received finasteride treatment for over ten years and exhibited a prostate volume reduction of less than 20%. This sample was designated as the Fin-P group based on the inclusion criterion, which required a reduction in prostate volume of less than 20% after at least 12 months of finasteride administration, and the exclusion criteria that encompassed concurrent usage of other pharmacological agents affecting prostate volume and presence or history of prostate cancer^[1]^. The remaining two samples were obtained from untreated BPH patients and labeled as the Fin-N group. Following ST sequencing, the FASTQ files were processed using spaceranger software (version 1.1.0) within the Space Ranger pipeline to generate feature barcode (FB) matrices. The FB matrices were then loaded into Seurat package (Version 3.0.0) in R for subsequent data filtering, normalization, integration, batch effects correction, dimensionality reduction, clustering, and visualization. Next, three scRNA-seq datasets (GSM5252458, GSM5252460, and GSM525246) originating from normal prostate tissues were downloaded from the NCBI Gene Expression Omnibus (GEO) database. These datasets were then loaded into Seurat package (Version 3.0.) for data filtering, normalization, integration, batch effects correction, dimensionality reduction, clustering, cell annotation, and visualization to serve as references for annotating clusters of ST data using robust cell type decomposition (RCTD) with doublet_mode set to 'full'. C2 and C3 clusters of ST data were then identified to be predominantly consisted of epithelial cells. Epithelial ST spots within C2 and C3 clusters were precisely selected using the well-established epithelial marker gene *EPCAM* for further analysis including evaluating finasteride treatment's impact on AR-dependent transcriptional activity, *SRD5A2* expression, luminal cell apoptosis and loss, basal cell proliferation, and enhanced cell communication between luminal cells and basal cells through TGFB1_EGFR pairs.

Additionally, four scRNA-seq datasets (GSM5252136, GSM5252137, GSM5252126, and GSM5252132) obtained from finasteride-treated (Fin-P) and untreated (Fin-N) BPH tissues, downloaded from the NCBI GEO database, were employed to confirm the results of ST data after being loaded into R package Seurat (Version 3.0.0) for data filtering, normlization, data integration, batch effects mitigation, dimensionality reduction, clustering, cell annotation, and visualization.

The aforementioned analyses were conducted using R software (Version 4.1.2). Batch effects were mitigated using the Harmony method. The FindAllMarkers function was employed to analyze the distinctive genes of each cluster. The CellCycleScoring function was used to calculate the G2M score and S score. Single-cell gene set enrichment analysis (ssGSEA) on Hallmark gene-set collection was performed using R package irGSEA (Version 1.1.3). Monocle2 was utilized to determine lineage differentiation of cell subtypes exhibiting potential developmental relationships. Cellular interaction analysis between different cell types in each group was conducted using CellphoneDB based on scRNA-seq data.

**Patients and sample collection**

A total of three prostate tissue samples were obtained from different patients diagnosed with BPH through pathology and imaging at the QingPu Branch of Zhongshan Hospital in 2019. Written informed consent was obtained from all participants included in this study. This study was approved by the Ethical Committee of QingPu Branch of Zhongshan Hospital (Qingyi2020-24).

**Visium spatial transcriptomics**

Three BPH tissue samples were included. Each tissue sample was longitudinally sectioned into two fragments, with one fragment utilized for pathological diagnosis and the other employed for ST sequencing. ST sequencing was performed using the Visium platform from 10x Genomics. Cryosections from three optical coherence tomography (OCT)-embedded prostate tissue samples (D1, P2, and 24-1) were placed on Visium spatial slides. The mRNA bound by printed capture oligos with spatial barcodes on the slide was converted to cDNA, which was subsequently transferred from the slide for library preparation. Spatial libraries were constructed using the Visium Spatial Library Construction Kit (PN-1000184; 10x Genomics), following the manufacturer's instructions, and then sequenced using HiSeq X10 system (Illumina) with 150 bp paired-end reads. Table S1 provides a summary of patient demographics and data quality.

**Spatial transcriptomic data analysis**

The FASTQ files were processed using spaceranger software (version 1.1.0) within the Space Ranger pipeline to generate the FB matrices. FB matrices were loaded into the R package Seurat (Version 3.0.0) for subsequent analysis. After performing data filtering and normalization, the FB matrices were integrated using the FindIntegrationAnchors and IntegrateData functions (dims = 1:50). Subsequently, batch effects were mitigated utilizing the Harmony method. The FindAllMarkers function was utilized to analyze the distinctive genes of each cluster (latent.vars = "nFeature_RNA", test.use = "MAST"). The CellCycleScoring function was employed to compute the G2M score and S score, enabling the assessment of cell cycle progression for each individual spot.

ssGSEA on Hallmark gene-set collection (N = 50) was performed using R package irGSEA (Version 1.1.3) (method = c ("AUCell", "UCell", "singscore", "ssgsea"). The rank aggregation algorithm (RRA) was used for comprehensively evaluation to screen out the significantly enriched gene sets in the results obtained by most gene set enrichment analysis methods.

**Single-cell RNA-sequencing data analysis**

The published single-cell RNA sequencing (scRNA-seq) datasets (GSM5252458, GSM5252460, GSM5252462, GSM5252136, GSM5252137, GSM5252126, and GSM5252132) were obtained from the NCBI GEO database for analysis ^[2]^. Among these datasets, three scRNA-seq datasets obtained from normal prostate tissues were utilized as a reference to annotate clusters of ST data. Additionally, four scRNA-seq datasets obtained from finasteride-treated (Fin-P) and untreated (Fin-N) BPH tissues were employed to investigate the mechanism underlying the development of finasteride treatment in human BPH. The data were loaded into the R package Seurat (Version 3.0.0) for subsequent analysis, and low-quality cells were excluded. Subsequent processes, including data normalization, integration (dims = 1:50), batch effect removal, and cell cycle assessment, followed the aforementioned ST analysis protocol. Cell annotation was performed based on differentially expressed genes (DEGs), cell feature gene set scores ^[3]^, and well-established cell markers.

**Cell type decomposition**

To determine the spatial distribution of cell groups, we utilized the integrated scRNA-seq dataset obtained from normal prostate tissue samples of three young organ donors (GSM5252458, GSM5252460, and GSM525246) as a reference to perform cell type decomposition within histological structures of each ST slide using RCTD with doublet_mode set to 'full' ^[^^4]^. Dentate weights were utilized to assess the cellular ratio at each ST spot.

**Trajectory analysis**

Monocle2 was utilized to ascertain the lineage differentiation of cell subtypes exhibiting potential developmental relationships. DDRTree was employed for inferring tree-like trajectories.

**Mapping the cellular interactions**

The cellular interaction analysis between different cell types in each group was performed using CellphoneDB ^[5]^, based on scRNA-seq data. Specific ligand-receptor (L-R) interaction pairs were identified as those that exhibited significant expression in the Fin-P samples but not in the Fin-N samples. A *P* value of less than 0.05 was considered statistically significant.

**Gene set scoring**

AddModuleScore method ^[3]^ and Gene Set Enrichment Analysis (GSEA) were employed to assess changes in the expression levels of both AR-mediated transcriptional gene set ^[6]^, Hallmark Androgen Response gene set, Hallmark Apoptosis gene set, and GOBP Cell Cycle gene set. G2M score and S score were calculated by using the CellCycleScoring function.

**Statistics**

The analysis was performed using R software (Version 4.1.2). *P*-values for the comparison between two variables were determined using a two-sided Wilcoxon rank-sum test. *P*-values for the correlation between two variables were determined using a Spearman test. Error bars represent standard error. A significance level of *P* < 0.05 was considered statistically significant and denoted as "*". "**" indicated a significance level of *P* < 0.01, while "***" indicated a significance level of *P* < 0.001, and "****" indicated a significance level of *P* < 0.0001.

**References**

[1] McConnell, J.D., Bruskewitz, R., Walsh, P., Andriole, G., Lieber, M., Holtgrewe, H.L., *et al*. The effect of finasteride on the risk of acute urinary retention and the need for surgical treatment among men with benign prostatic hyperplasia. Finasteride Long-Term Efficacy and Safety Study Group. *N. Engl. J. Med*. **338**(9), 557 (1998) .

[2] Joseph, D.B., Henry, G.H., Malewska, A., Reese, J.C., Mauck, R.J., Gahan, J.C. *et al*. Single-cell analysis of mouse and human prostate reveals novel fibroblasts with specialized distribution and microenvironment interactions. *J. Pathol.* **255**(2), 141-154 (2021).

[2] Henry, G.H., Malewska, A., Joseph, D.B., Malladi, J. Lee, V.S., Torrealba, J. *et al*. A Cellular Anatomy of the Normal Adult Human Prostate and Prostatic Urethra. *Cell Rep.* **25**(12), 3530-3542.e5 (2018).

[3] able, D.M., Murray, E., Zou, L.S., Goeva, A., Macosko, E.Z., Chen, F. *et al*. Robust decomposition of cell type mixtures in spatial transcriptomics. *Nat Biotechnol.* **40**(4), 517-526 (2022).

[4] Vento-Tormo, R., Efremova, M., Botting, R.A., Turco, M.Y., Vento-Tormo, Meyer, M., K.B. *et al*. Single-cell reconstruction of the early maternal-fetal interface in humans. *Nature* **563**(7731), 347–353 (2018).

[5] Qiu, X., Boufaied, N., Hallal, T., Feit, A., de Polo, A., Luoma, A.M. *et al*. MYC drives aggressive prostate cancer by disrupting transcriptional pause release at androgen receptor targets. *Nat. Commun.* **13**(1), 2559 (2022).

**Supplementary Tables**

**Supplementary Tab. S1. General information of patients with BPH and the quality of their ST data**

| ID (Patients) | Age | Finasteride treatment | *Prostatic volume (mm) | tPSA | ID (Sample) | ID (Group) | Number of Spots | Median Genes/spot | Median UMI/spot | Q30 Bases in UMI |
| --- | --- | --- | --- | --- | --- | --- | --- | --- | --- | --- |
| D1 | 88 | No | 48*52*40 | 2.824 | D1 | Fin-N | 2438 | 3334 | 8358 | 92.70% |
| P2 | 60 | No | 54*49*46 | 3.2 | P2 | Fin-N | 2549 | 2541 | 5626 | 92.80% |
| 24-1 | 88 | Yes (>10 years) | 52*38*51 | 4.99 | 24-1 | Fin-P | 2388 | 2684 | 6002 | 95.10% |

*Prostate volume was detected before surgery by ultrasonic examination.

**Supplementary Figures**


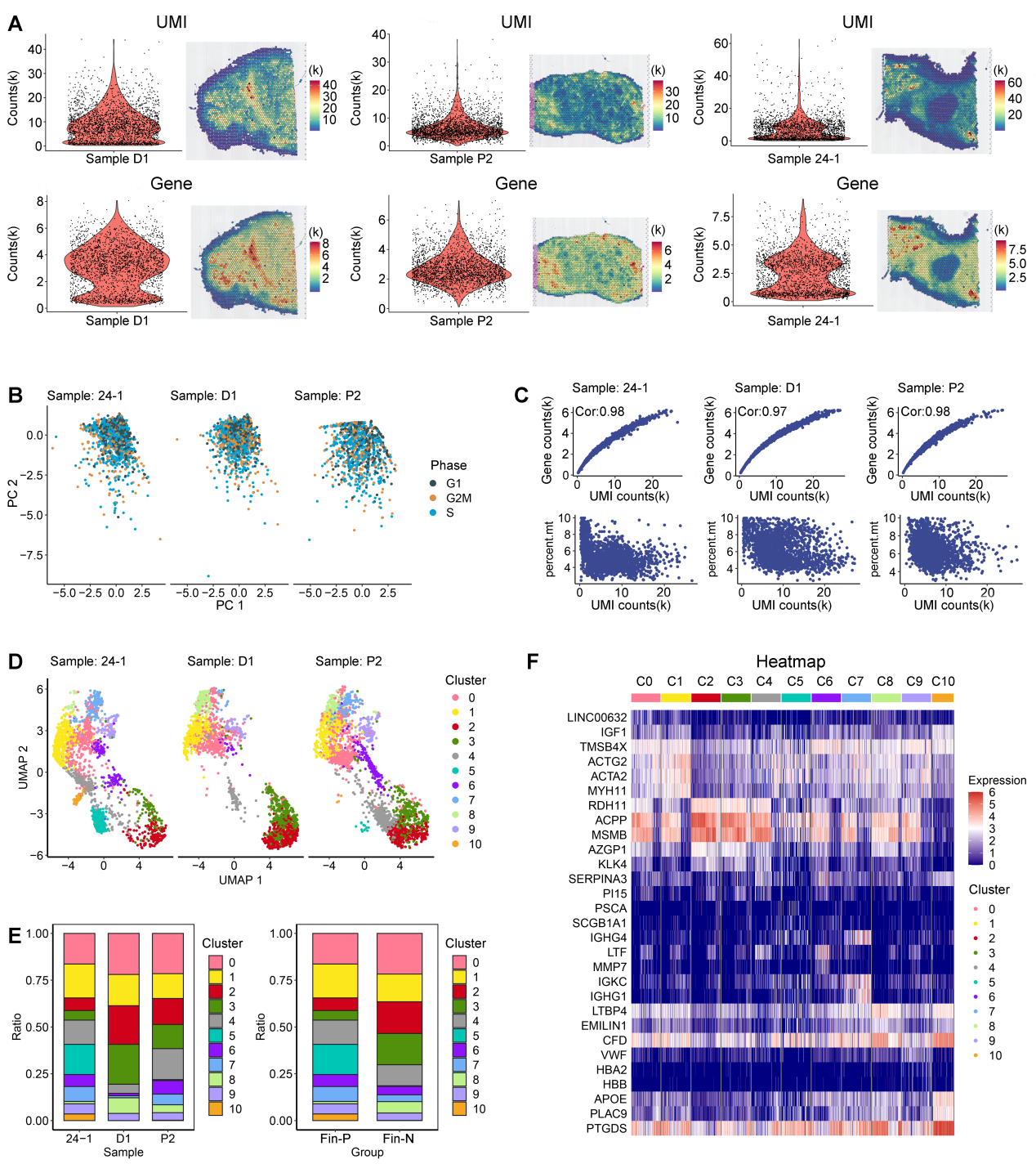


**Supplementary Fig. S1.** **Quality assessment and comprehensive overview of ST data. (A)** nCount (UMI) and nFeature (gene) per spot. **(B)** Principal Component Analysis (PCA) plots depicting the cell cycle dynamics. **(C)** Correlation between nFeature and nCount (top); Correlation between percent.mt and nCount (bottom). **(D)** UMAP of 11 clusters in each sample. **(E)** Ratio of clusters in each sample and group. **(F)** Heatmap illustrating the top three differentially expressed genes (DEGs) within each cluster.


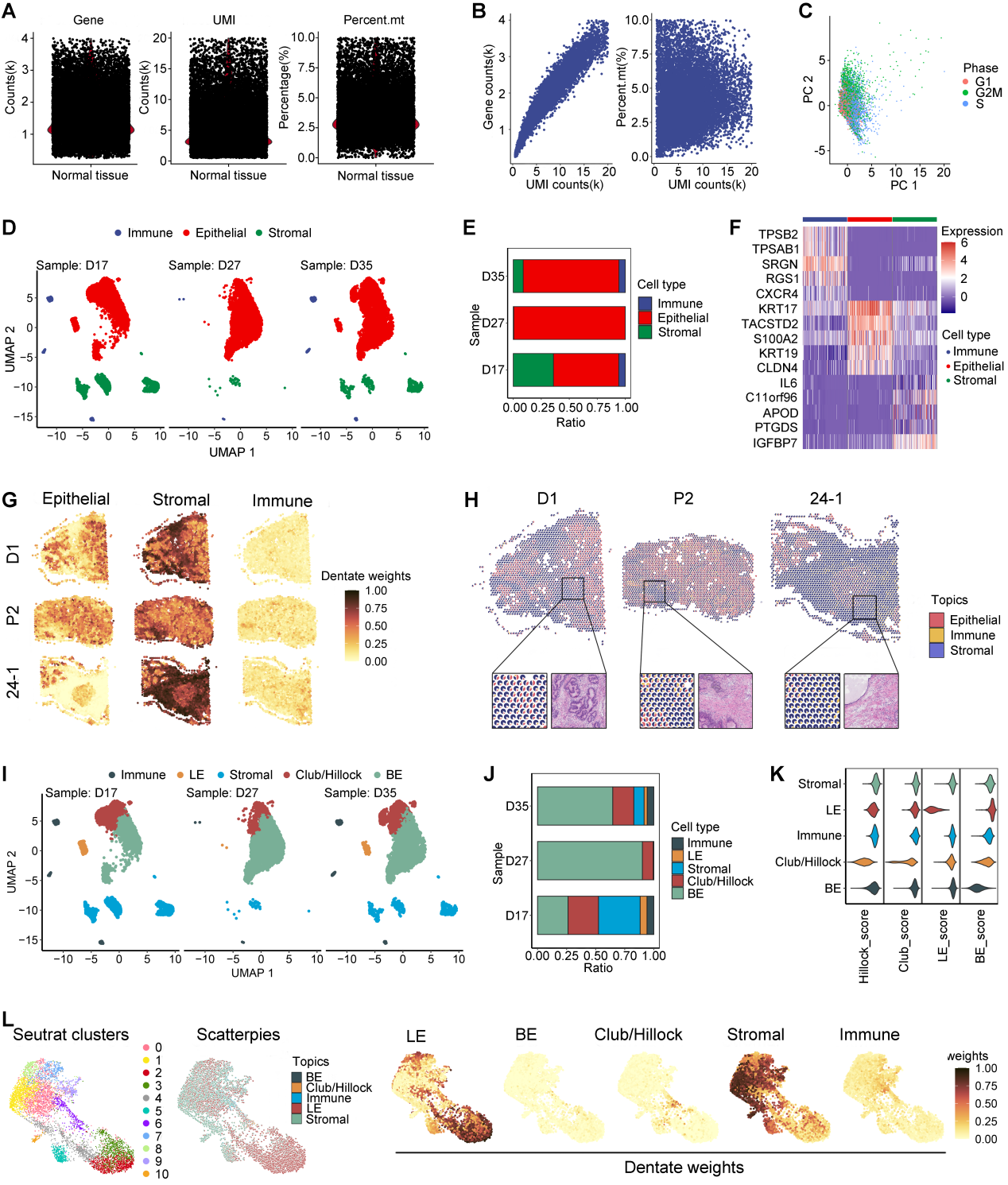


**Supplementary Fig. S2. RCTD of ST data. (A-C)** Quality assessment and comprehensive overview of scRNA-seq data (reference): **(A)** UMIs, genes, and percent.mt per spot; **(B)** Correlation between genes and UMIs, as well as correlation between percent.mt and UMIs; **(C)** PCA plots depicting the cell cycle dynamics. **(D-F)** Preliminary cell typing for scRNA-seq data (reference): **(D)** UMAP coloured by cell types; **(E)** Ratio of cell types in each sample; **(F)** Heatmap illustrating the top five DEGs within each cell type. **(G****)** Dentate weights and **(H)** scatterpies of epithelial, stromal, and immune cells in each tissue slide. **(I-K)** Cell typing for scRNA-seq data (reference): **(I)** UMAP coloured by cell types; **(J)** Ratio of cell types in each sample; **(K)** Violin plot of cell feature scores in each cell type. **(L)** UMAP coloured by 11 clusters of ST data, UMAP of the scatterpies, and UMAP coloured by the dentate weights of LE, BE, Club/Hillock, stromal, and immune cells.


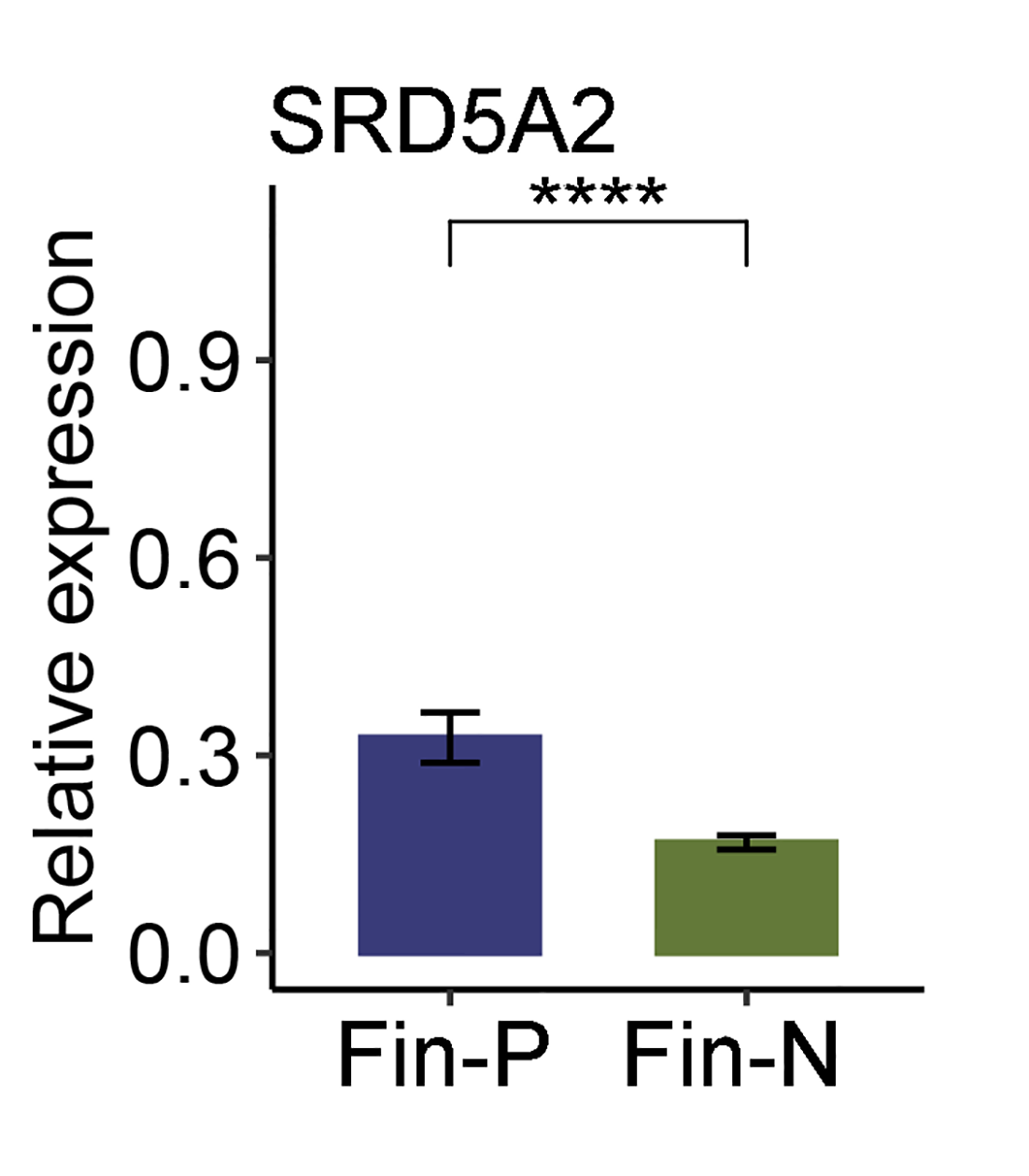


**Supplementary Fig. S3. Bar plots depicting the gene expression level of *SRD5A2* in the Fin-P and Fin-N ST epthelia spots**

**
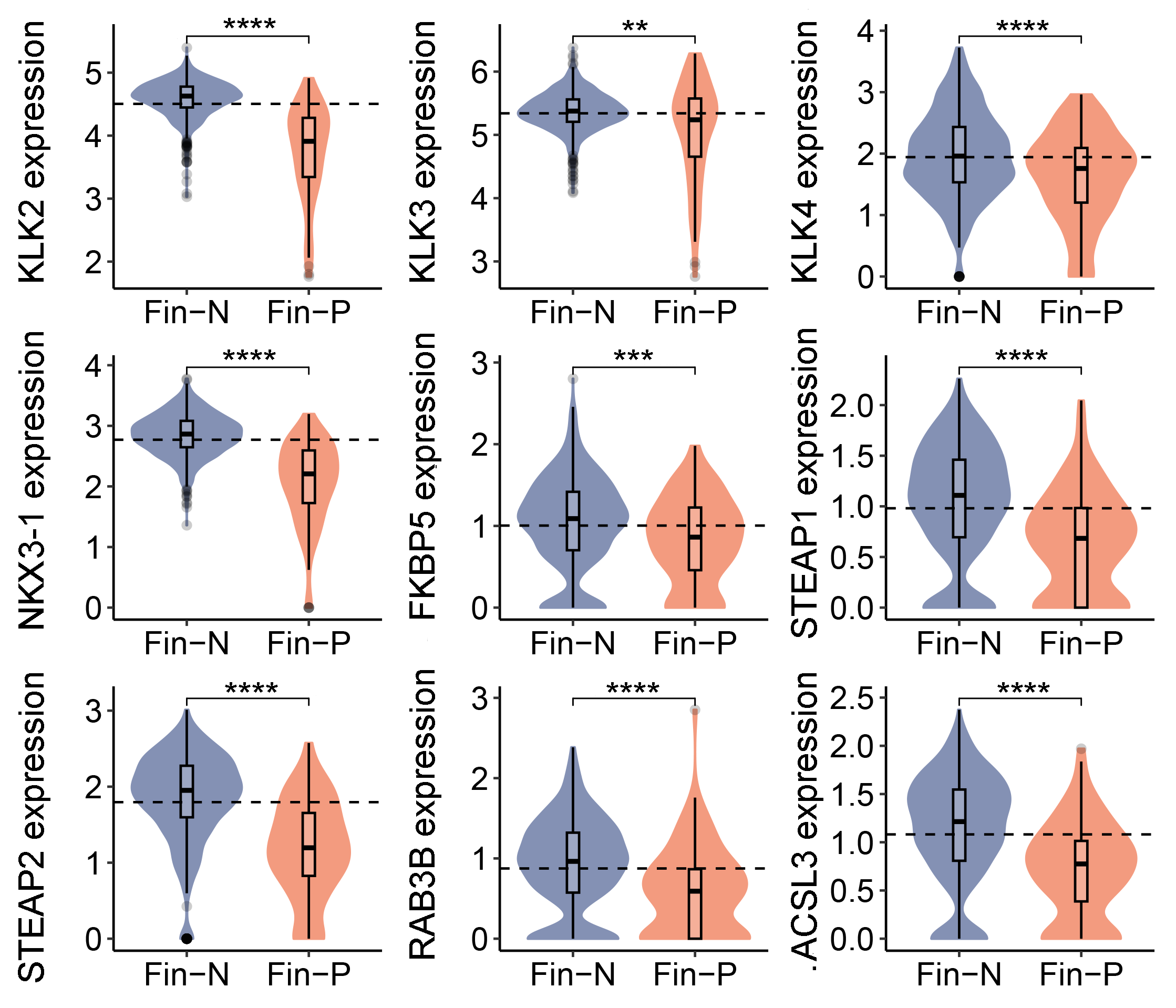
**

**Supplementary Fig. S4. Violin plots depicting the expression levels of *KLK2*, *KLK3*, *KLK4*, *NKX3-1*, *FKBP5*, *STEAP1*, *STEAP2*, *RAB3B*, and *ACSL3* in Fin-P and Fin-N epithelial ST spots.**

**
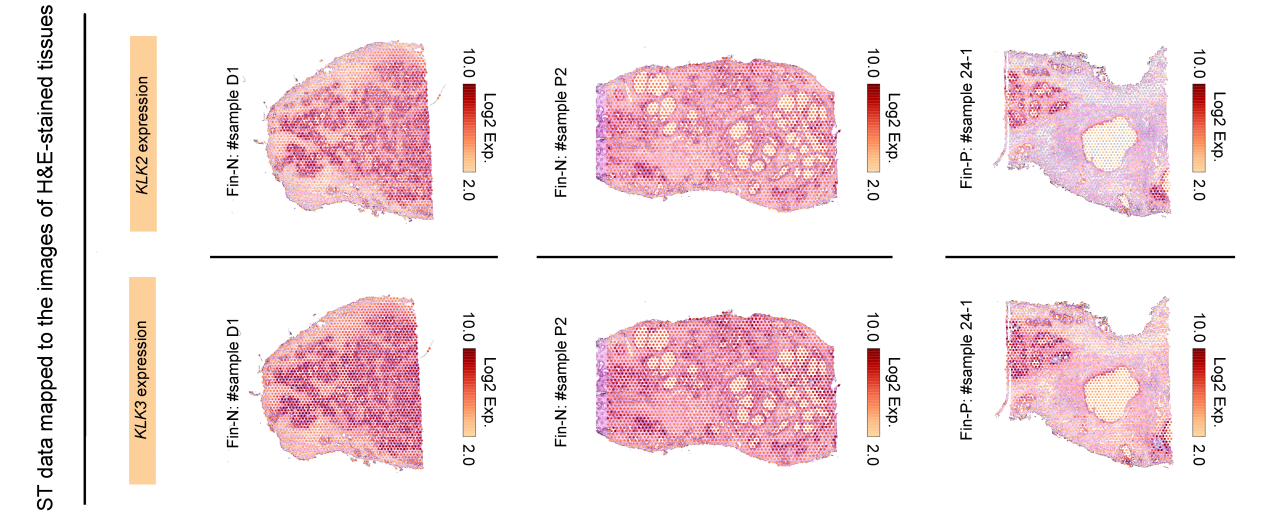
**

**Supplementary Fig. S5. The expression of *KLK2* and *KLK3* mapped to the images of H&E-stained tissues.** Scale bars, 1 mm.

**
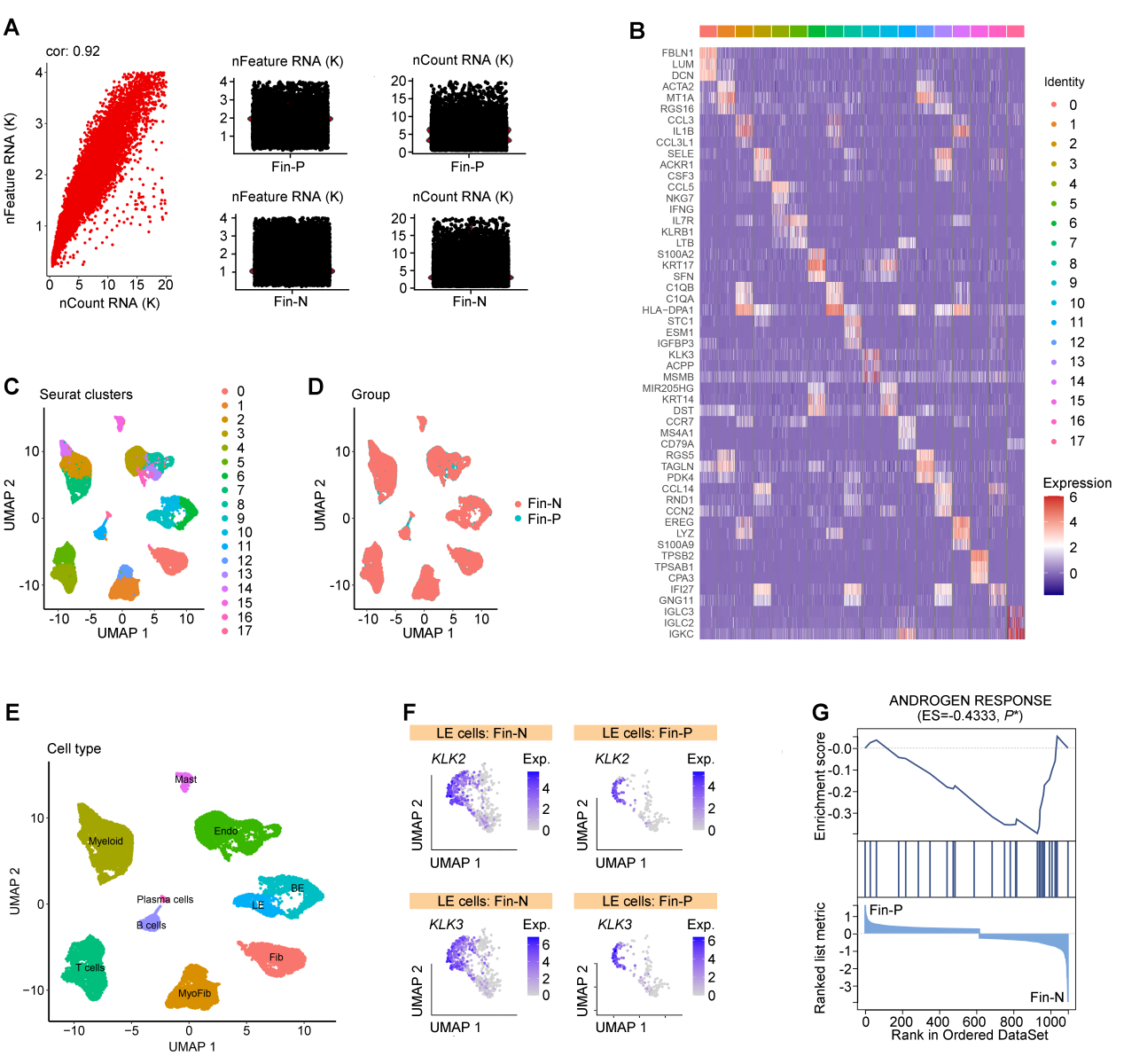
**

**Supplementary Fig. S6. Comprehensive overview of BPH scRNA-seq data. (A)** UMIs, genes, and percent.mt per spot; Correlation between genes (nFeature RNA) and UMIs (nCount RNA). **(B)** Heatmap illustrating the top three DEGs within each cluster. **(C)** UMAP colored by clusters. **(D)** UMAP colored by groups. **(E)** UMAP visualization displaying distinct cell types using a diverse color palette. **(F)** UMAP of LE cells colored by the expression of *KLK2* and *KLK3*. **(G)** GSEA plot interpreting changes in the expression levels of Hallmark Androgen Response gene set across Fin-P and Fin-N LE cells.

**
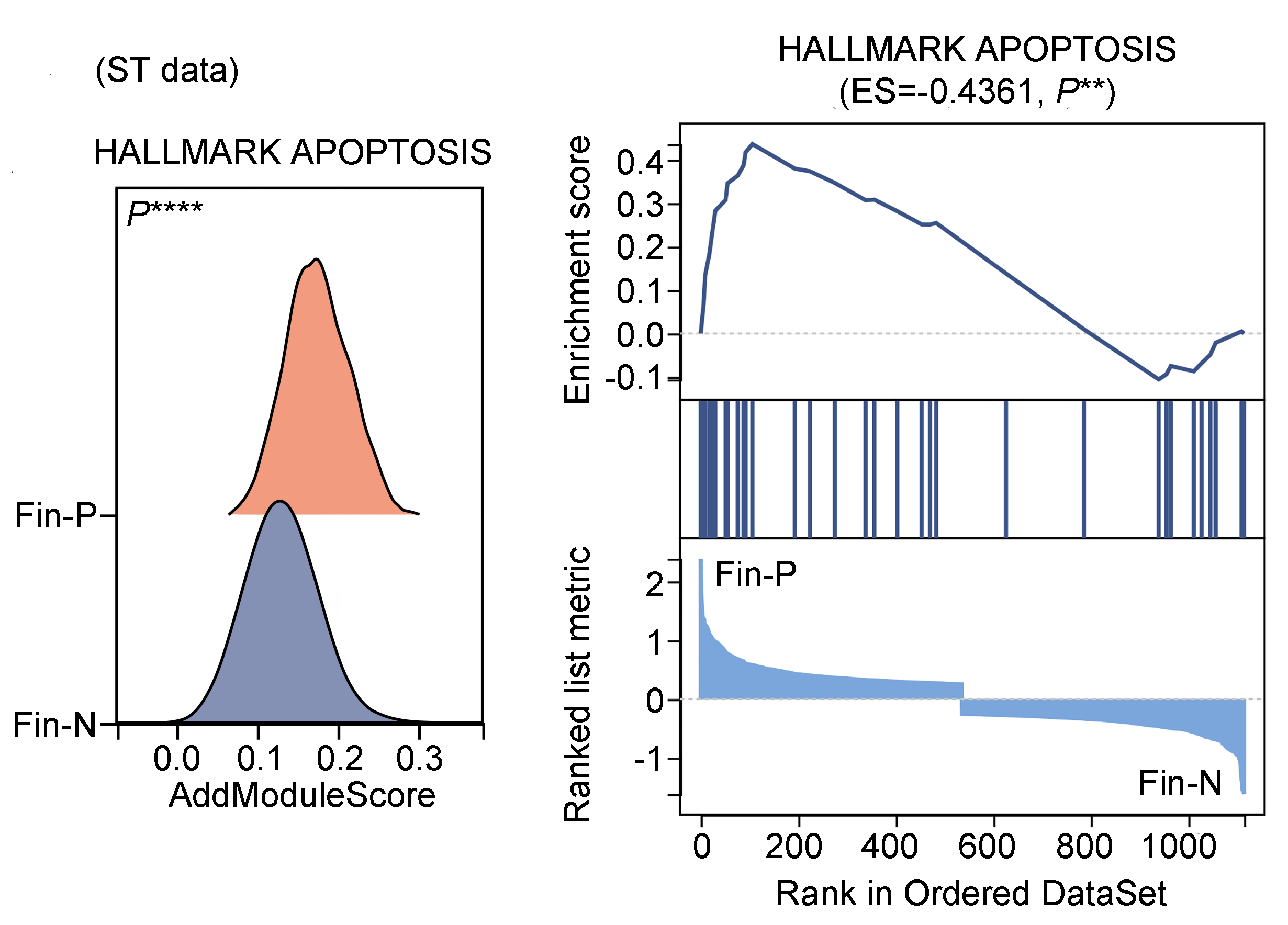
**

**Supplementary Fig. S7. Ridge plot depicting the scores of Hallmark Apoptosis gene set in Fin-P and Fin-N epithelial ST spots; GSEA plot interpreting changes in the expression levels of Hallmark Apoptosis gene set across Fin-P and Fin-N epithelial ST spots.**

**
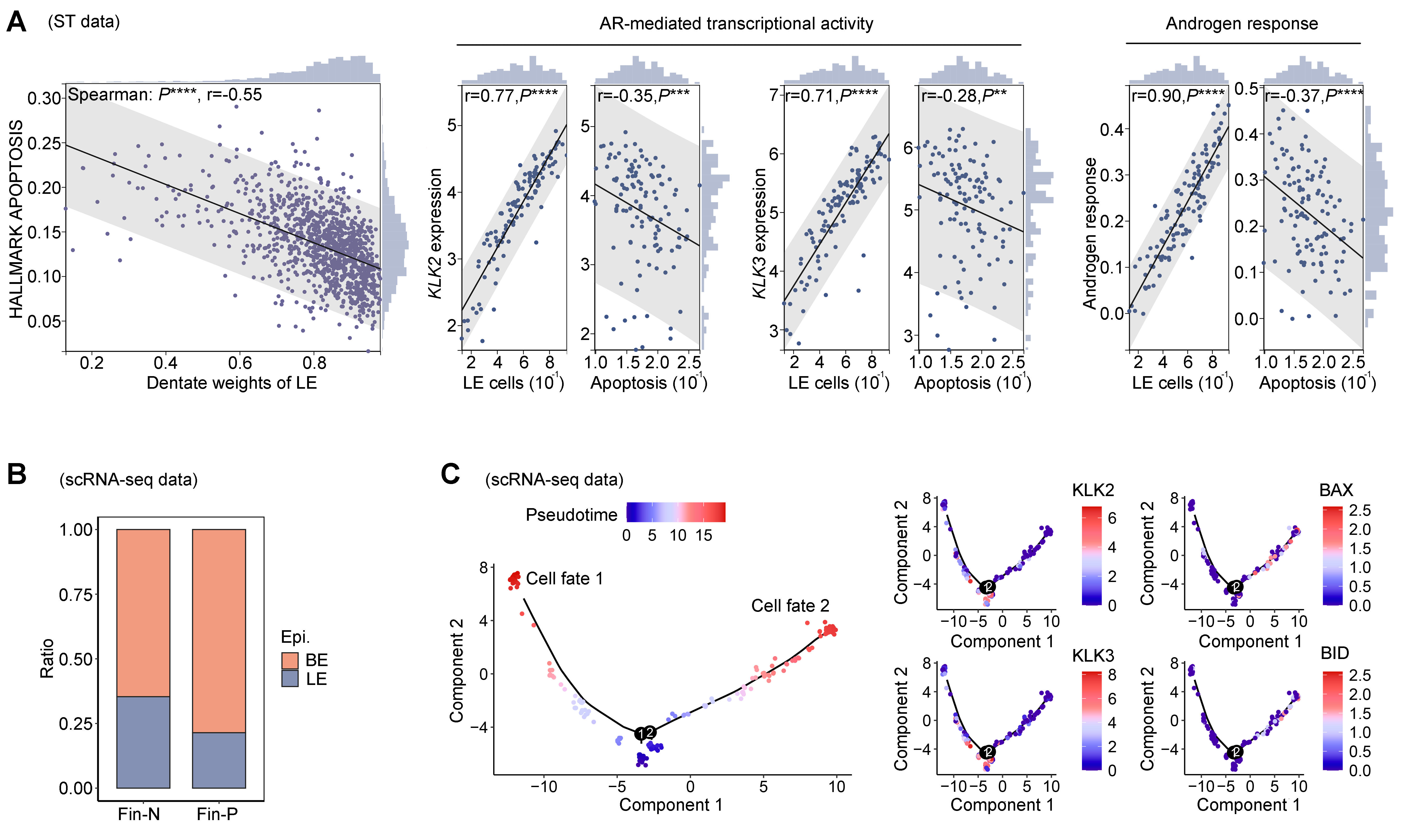
**

**Supplementary Fig. S8. An attenuation of AR-mediated transcriptional activity leading to LE cell loss in the Fin-P BPH epithelia. (A)** Scatter plots illustrating the Spearman correlations in epithelial ST spots. **(B)** Bar plot depicting the ratio between LE cells and BE cells in scRNA-seq data. **(C)** Pseudo-time cell trajectory of LE cells colored by the pseudo-time and the expression levels of *KLK2*, *KLK3*, *BAX* and *BID*.

**
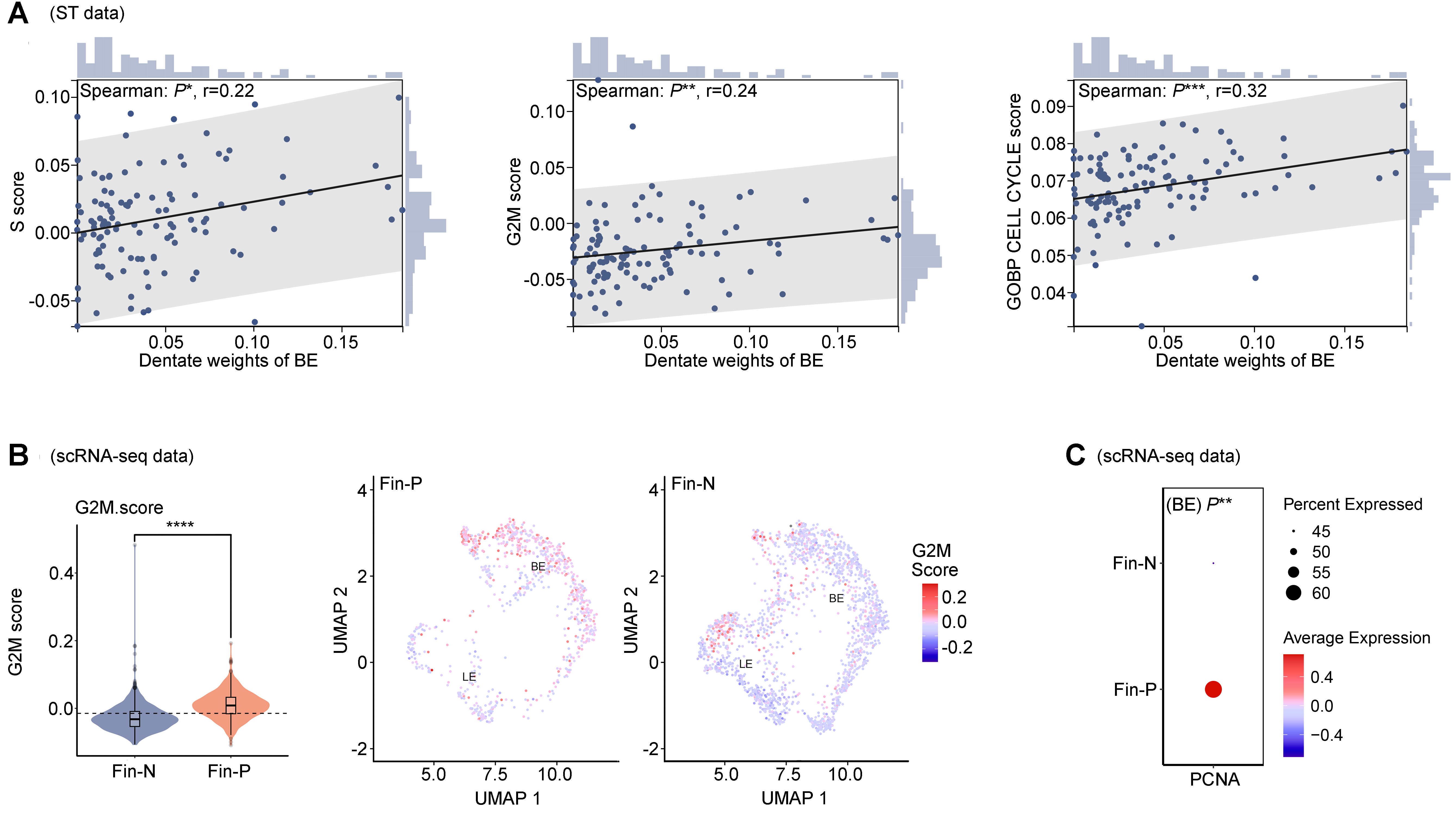
**

**Supplementary Fig. S9. Augmented BE cell proliferation in Fin-P BPH epithelia. (A)** Scatter plots illustrating the Spearman correlations in epithelial ST spots. **(B)** scRNA-seq data: Violin plot depicting the G2M score of BE cells; UMAP of BE and LE cells colored by G2M score. **(C)** scRNA-seq data: Dot plots illustrating the expression of *PCNA* in BE cells.


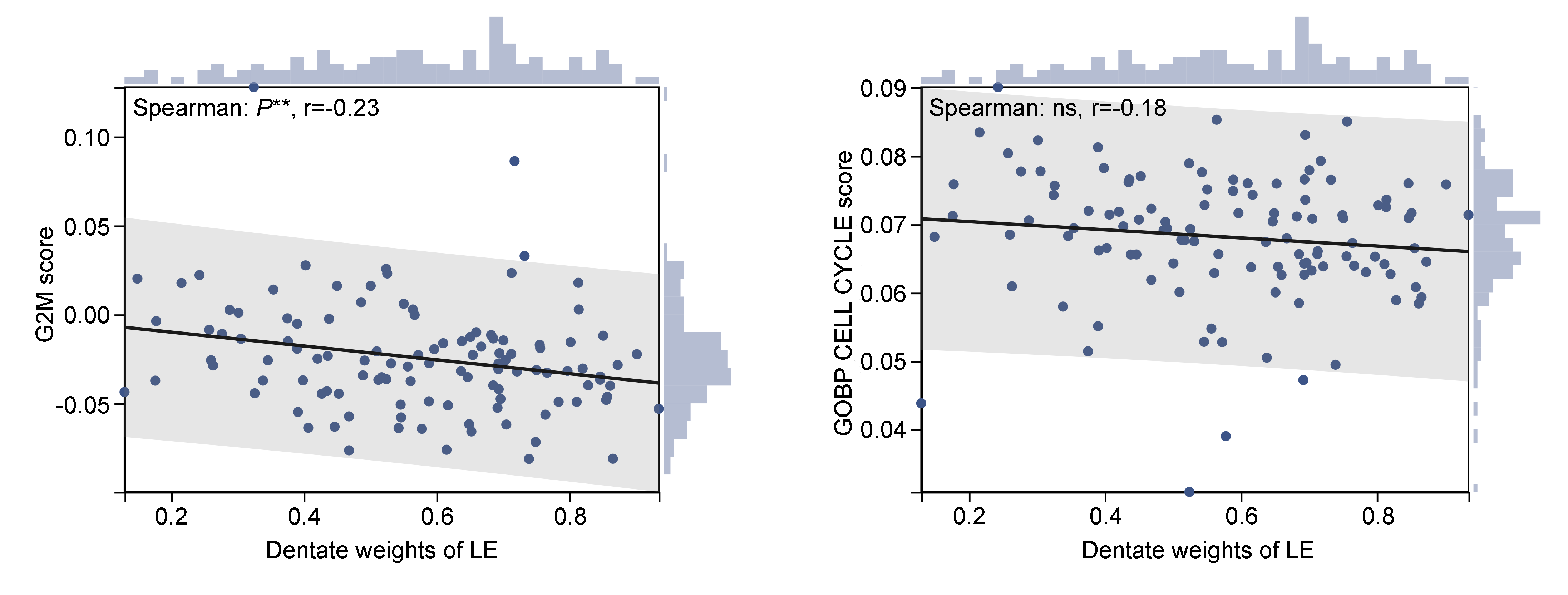


**Supplementary Fig. S10. Scatter plots illustrating the Spearman correlations.**

**
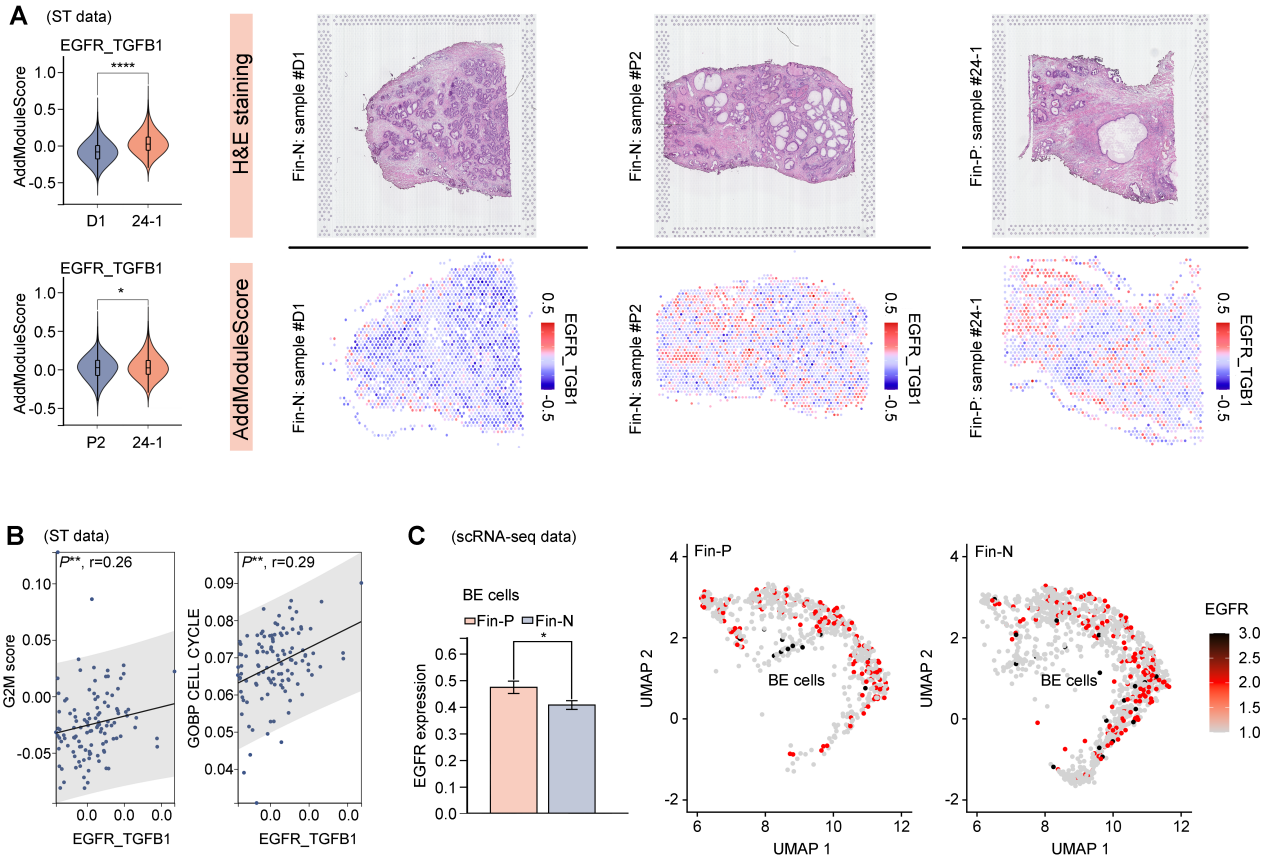
**

**Supplementary Fig. S11. EGFR playing positive role in facilitating BE proliferation. (A****)** Violin plot depicting the co-expression score of *TGFB1* and *EGFR* in Fin-P and Fin-N BPH tissue ST spots; Images depicting H&E-stained sections of BPH tissues and BPH tissue sections coloured by the co-expression score of *TGFB1* and *EGFR*. **(B)** Scatter plot illustrating the Spearman correlations in Fin-P epithelial ST spots. **(C)** Bar plot and UMAPs depicting the *EGFR* expression of BE cells in scRNA-seq data obtained from Fin-P and Fin-N BPH patients.
